# Multimodality Imaging in the Study of the Left Atrium

**DOI:** 10.3390/jcm11102854

**Published:** 2022-05-18

**Authors:** Sergio Moral, Marc Abulí, Pau Vilardell, Emilce Trucco, Esther Ballesteros, Ramon Brugada

**Affiliations:** 1 Cardiology Department, Hospital Universitari Doctor Josep Trueta, 17007 Girona, Spain; marcabuli54887@gmail.com (M.A.); pau.vilardell.rigau@gmail.com (P.V.); emilcetrucco@gmail.com (E.T.); ramon@brugada.org (R.B.); 2 Dirección Territorial de Radiologia i Medicina Nuclear de Girona, Insititut de Diagnòstic per la Imatge (IDI), Institut D’Investigació Biomèdica de Girona (IDIBGI), 17007 Girona, Spain; esther.ball@gmail.com; 3 Cardiovascular Genetics Centre, University of Girona-IDIBGI, 17190 Girona, Spain; 4 Medical Science Department, School of Medicine, University of Girona, 17004 Girona, Spain; 5 Centro Investigación Biomédica en Red, Enfermedades Cardiovasculares (CIBERCV), 28029 Madrid, Spain

**Keywords:** left atrium, transthoracic echocardiography, cardiac magnetic resonance, multidetector computed tomography, electroanatomical maps

## Abstract

The left atrium (LA) plays a vital role in maintaining normal cardiac function. Many cardiac diseases involve the functioning of the LA directly or indirectly. For this reason, the study of the LA has become a priority for today’s imaging techniques. Assessment of LA size, function and wall characteristics is routinely performed in cardiac imaging laboratories when a patient undergoes transthoracic echocardiography. However, in cases when the LA is the focus of disease management, such as in atrial fibrillation or left atrial appendage closure, the use of multimodality is critical. Knowledge of the usefulness of each cardiac imaging technique for the study of LA in these patients is crucial in order to choose the most appropriate treatment. While echocardiography is the most widely performed technique for its evaluation and the study of wall deformation analysis is increasingly becoming more reliable, multidetector computed tomography allows a detailed analysis of its anatomy to be carried out in 3D reconstructions that help in the approach to interventional treatments. In addition, the evaluation of the wall by cardiac magnetic resonance imaging or the generation of electroanatomical maps in the electrophysiology room have become essential tools in the treatment of multiple atrial pathologies. For this reason, the goal of this review article is to describe the basic anatomical and functional information of the LA as well as their study employing the main imaging techniques currently available, so that practitioners specializing in cardiac imaging techniques can use these tools in an accurate and clinically useful manner.

## 1. Introduction

The anatomy and functionality of the left atrium (LA) have prognostic implications in multiple cardiological pathologies [1,2,3,4]. In recent years, with the evolution of imaging techniques, the study of the LA has become essential for a complete assessment of the diagnosis, prognosis and indications for treatment in cardiological patients [5,6,7,8,9]. Although its main role has been described in atrial fibrillation (AF) and mitral valve disease, its implications are increasingly relevant in cardiomyopathies, diastolic dysfunction and ischemic heart disease, among others [5,6,7,8]. Atrial involvement has become so important that some authors advocate the definition of Atrial Cardiomyopathies as “any complex of structural, architectural, contractile or electrophysiological changes affecting the atria with the potential to produce clinically relevant manifestations” [9].

Atrial study has classically been based on transthoracic (TTE) and transesophageal echocardiography (TEE) [10,11]. However, the development of other techniques, such as cardiac magnetic resonance (CMR) imaging and multidetector computed tomography (MDCT), has helped to increase our knowledge of its anatomy and functionality [12]. Moreover, the generation of atrial electroanatomic maps in the electrophysiology laboratory has provided better treatment of arrhythmias with atrial origin [9].

The goal of this review article is to describe the basic anatomical and functional information of the LA as well as their study employing the main imaging techniques currently available, so that practitioners specializing in cardiac imaging techniques can use these tools in an accurate and clinically useful manner.

## 2. Anatomy and Functions of the LA

**Figure 1:**
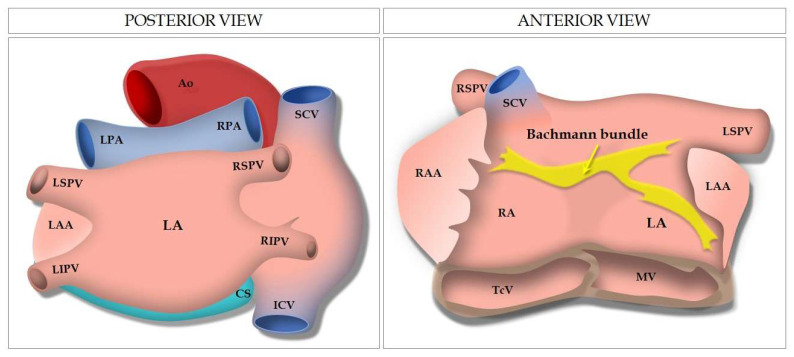
Illustration showing the anterior and posterior view of the LA and the anatomical relationships with adjacent structures. Ao: aorta; CS: coronary sinus; ICV: inferior cava vein; LA: left atrium; LAA: left atrial appendage; LIPV: left inferior pulmonary vein; LPA: left pulmonary artery; LSPV: left superior pulmonary vein; MV: mitral valve; RA: right atrium; RAA: right atrial appendage; RIPV: right inferior pulmonary vein; RPA: right pulmonary artery; RSPV: right superior pulmonary vein; SCV: superior cava vein; TcV: tricuspid valve.

The LA is a thin-walled structure located in the inflow path from the pulmonary veins (PVs) to the left ventricle (LV) and is characterized by a main body and a finger-like trabeculated appendage [13]. Left atrial appendage (LAA) is the only remnant of the original embryonic LA, whereas the main smooth-walled left atrial body develops later from the outgrowth of the PVs [14]. The LA posterior wall is the anatomical location of the venous component and contains the inflow of the four PVs (Figure 1). The myoarchitecture of the LA is highly complex and with great variability between individuals, with overlapping and differently oriented myofibers that are not divided into layers by sheaths or fibrous laminae [15,16]. Nevertheless, some structural muscular components are regularly constant, such as Bachmann’s bundle, the most superficial group of myofibers which run along the LA anterior wall parallel to the atrioventricular groove [13].

The LA has three main functions: neurohormonal, regulatory and mechanical. The neurohormonal function is related to atrial secretion of atrial natriuretic peptide, resulting essentially in diuresis and vasodilation. This secretion occurs especially with dilation or stretching of the atrial wall [17]. The regulatory function is based on the actions performed by mechanoreceptors located in the venous–atrial junctions [18]. These atrial receptors are highly efficient, and fluctuation in venous volume of <1% can be signaled to the brain through autonomic nerves, mandatory in situations of difficult control of blood volume such as hemorrhage or heart failure [19].

**Figure 2:**
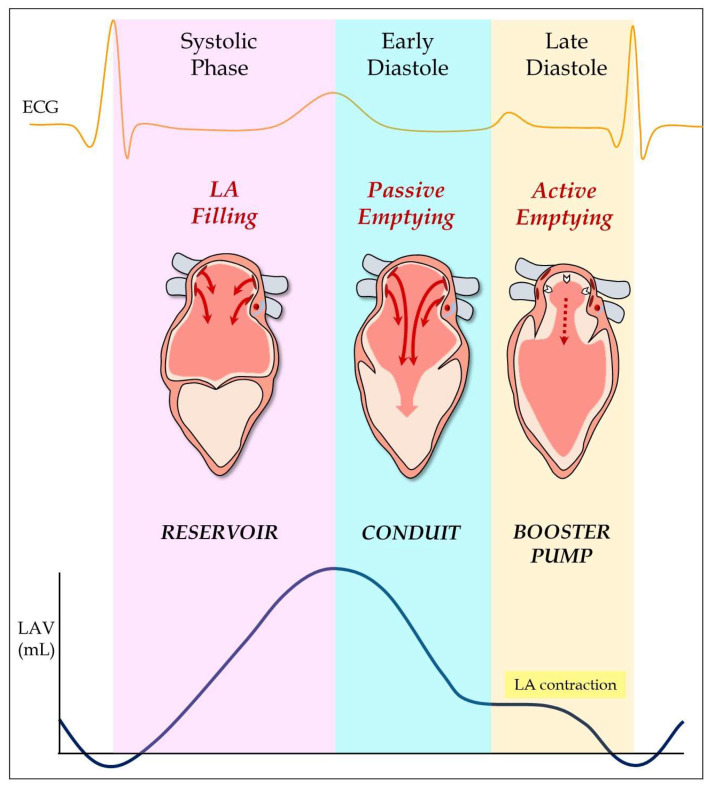
Mechanical function of the LA showing the three different phases during the cardiac cycle: left atrial filling, passive emptying and active emptying.

Finally, the mechanical function of LA is usually defined by three phases during the cardiac cycle (Figure 2). The reservoir phase takes place during ventricular systole when the LA collects blood coming from the PVs. This phase is determined by LA relaxation during LV contraction, which implies an increase in the atrial volume [20]. In the early diastole, the conduit phase occurs, which is the passive emptying of the atrium into the LV due to the opening of the mitral valve with a consequent decrease in atrial pressure. At end-diastole, atrial contraction occurs (booster pump phase), which represents the active emptying of the LA [21,22]. These phases can be altered in many pathologies, especially in arrhythmias affecting the LA, such as AF.

Although the three atrial functions are relevant for the cardiovascular system, from the point of view of imaging techniques, it is the mechanical one and its phases that can best be studied.

## 3. Multimodality Imaging of the LA Echocardiography

### 3.1. TTE

TTE is the first-choice technique for both anatomical and functional analysis of the LA, because of its wide accessibility, rapidity of assessment and the considerable data it provides. Different parameters have been described for LA evaluation with TTE, from M-mode to the latest 3D technologies (Table 1; [23,24,25,26]). However, the lack of software available in most centers for 3D TTE assessment have restricted its use in routine clinical practice.

**Figure 3:**
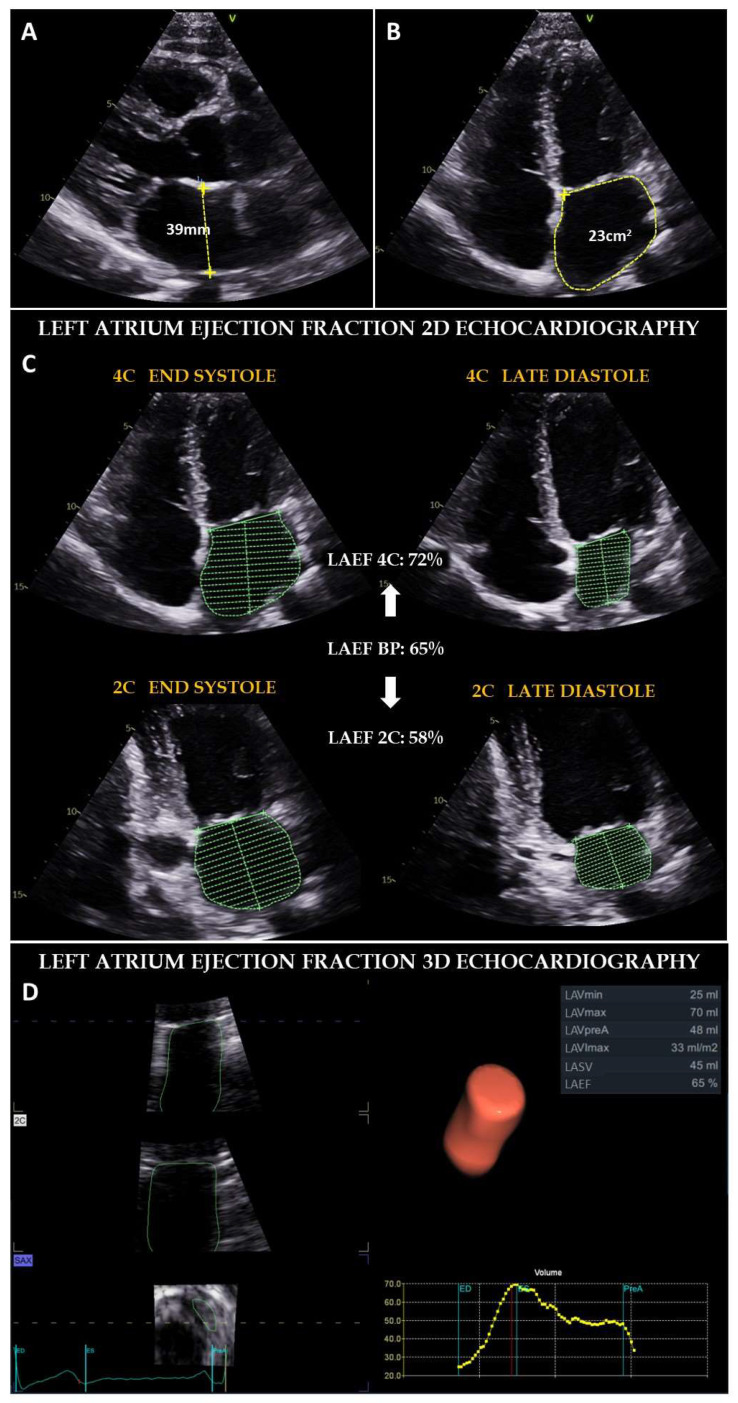
Methods for the determination of LA size and function by performing TTE. (**A**) Maximal anteroposterior LA diameter measured in 2 dimensions in parasternal long axis view from leading edge to leading edge, perpendicular to the posterior wall of LA. (**B**) Maximal LA area measured in apical four chamber view. (**C**) Total LA ejection fraction calculated by 2 dimensions in four and two chamber apical view. Maximum volume is assessed in end systole just before mitral valve opening. Minimum volume is estimated in late diastole in the onset of the P wave. (**D**) Total LA ejection fraction measured in 3 dimensions, acquiring a complete volume including the LA and applying specific software for the calculation.

Anatomically, the anteroposterior atrial diameter has been the most widely accepted parameter for the evaluation of atrial size, whether calculated by M-mode or 2D modalities [23]. Nevertheless, it is currently recommended to perform the measurement of atrial area and/or volume using 2D and 3D modes, due to the higher accuracy in the study of this structure, because of the LA asymmetry (Figure 3). Furthermore, the indexing of atrial volume to patient body surface area is relevant for the prognostic information it provides in some pathologies, especially in the assessment of diastolic dysfunction [27].

**Figure 4:**
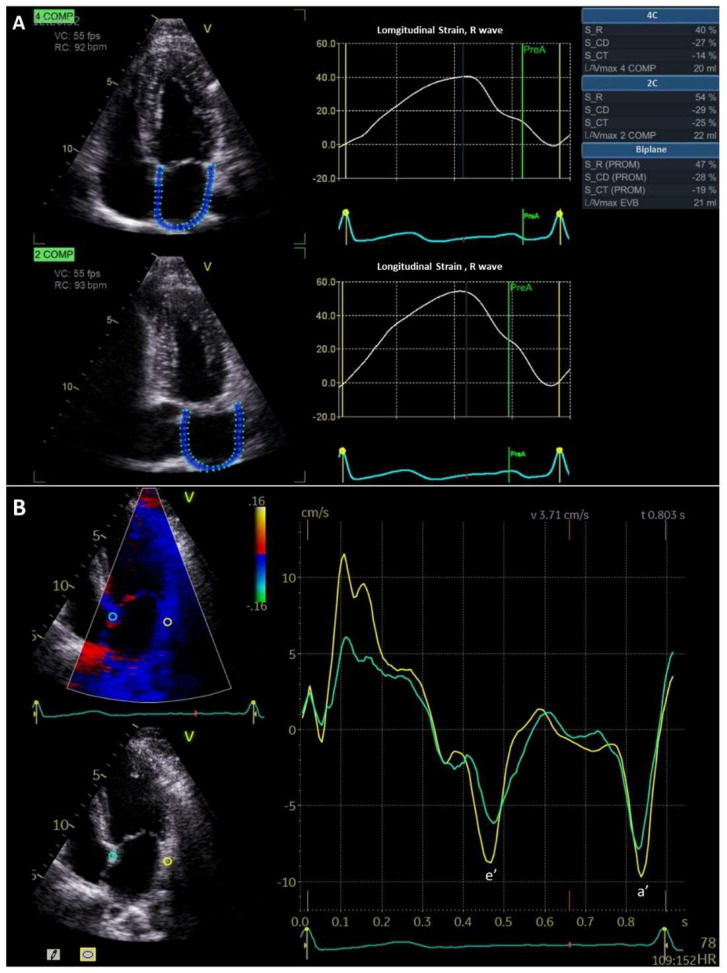
Atrial function evaluated by speckle tracking (**A**) and tissue Doppler imaging (**B**). Left atrial strain curves (**A**) with R wave as time reference, showing the 3 phases of the atrial function measured in apical four and two chamber views: reservoir longitudinal strain (47%) is the peak positive value, conduit longitudinal strain (−28%) is the measurement during early diastole and contractile longitudinal strain (−19%) is the value during end-diastole. Tissue Doppler imaging in the apical four chamber view to assess regional LA function (**B**). The samples are placed in atrial (green) and in the lateral wall (yellow). Peak diastolic velocities are assessed. Early diastolic filling is indicated by e’ and end-diastolic filling with a’.

Atrial functional evaluation by TTE is mainly based on the study of atrial contractility and wall deformation. The former can be calculated with 2D or 3D techniques from the atrial volumes in the different phases and the latter by speckle tracking or tissue Doppler imaging (Figure 3 and Figure 4). The formulae for the calculation of the three atrial volumetric phases are included in Table 1 [23,24,25,26,28]. However, given the great variability of these parameters between individuals and pathologies, they are rarely used in clinical practice and are mainly applied in research studies [29].

The assessment of atrial wall deformation is performed by calculating the strain and strain rate by segments and globally. Strain is the deformation of the wall and strain rate is myocardial deformity over time (the speed of myocardial deformation). These values are obtained from the three atrial phases if the patient is in sinus rhythm. Global strain is currently the most widely used, due to its lower variability in calculation [25]. Tissue Doppler imaging also allows estimation of these parameters in TTE. However, this depends on the angle of insinuation, and considerably limits its use in the atrium, because the analysis provided is mainly regional [30,31]. The speckle tracking technique is a post-processing algorithm that quantifies LA deformation by tracking the motion of speckles within the whole myocardium through the cardiac cycle [32]. It is an angle-independent measurement, which facilitates such calculations and has made it the reference technique for calculating atrial deformation.

The analysis of atrial deformation, particularly by speckle tracking, is proving to be of great clinical relevance in many pathologies, such as in AF for the assessment of the risk of relapses or embolisms, in infiltrative diseases such as amyloidosis, and in valvulopathies such as aortic stenosis or mitral insufficiency, where its impairment predicts a worse prognosis in these illnesses [33,34,35,36].

### 3.2. TEE

**Figure 5:**
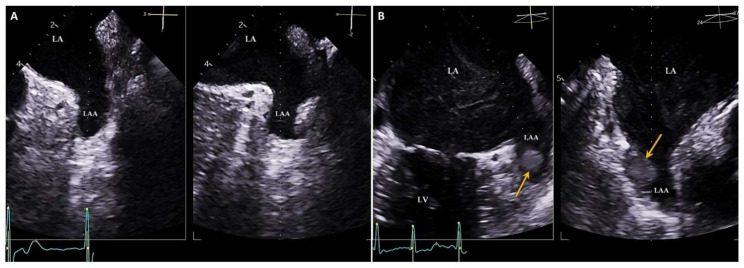
Analysis of LAA prior to electrical cardioversion with 2D TEE multiplane mid-esophageal view to assess the stroke risk. LAA without thrombus (**A**) and with thrombus inside (yellow arrows; (**B**)). LAA: Left atrial appendage; LA: Left atrium.

**Figure 6:**
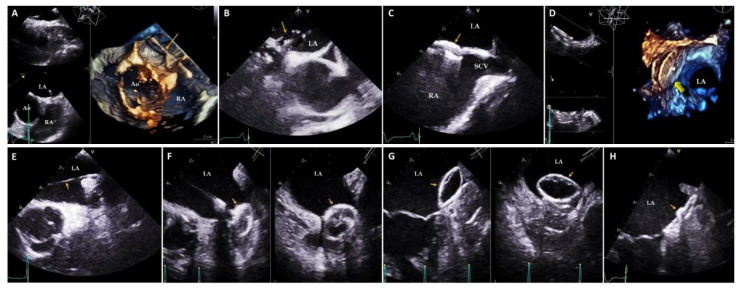
TEE images during invasive procedures in the hemodynamics laboratory. (**A**) 3D TEE image showing crossing of the guidewire (yellow arrow) through the atrial septum. (**B**) 2D TEE image demonstrating the inflated balloon (yellow arrow) through the patent foramen ovale for the sizing of the device to be implanted. (**C**) 2D TEE Midesophageal bicaval plane image depicting the patent foramen ovale closure device (yellow arrow) properly positioned. (**D**) 3D TEE image showing correct patent foramen ovale closure device (yellow arrow) placement from the left atrial view. (**E**) 2D TEE Midesophageal aortic valve short axis view illustrating the catheter (yellow arrow) in the LA resting on the left superior pulmonary vein. (**F**) 2D TTE multiplanar image demonstrating the lobe (yellow arrows) deployment of the atrial appendage closure device. (**G**) 2D TTE multiplanar image showing the implanted atrial appendage closure device (yellow arrows) while traction maneuvers are performed to assess the correct positioning of the device. (**H**) 2D TTE image showing the appendage closure device (yellow arrow) correctly positioned at the end of the procedure. Ao: Aorta; LA: Left atrium; RA: Right atrium; SCV: Superior cava vein.

TEE is usually performed to study some morphological characteristics of the LA. The main indications of the technique are the assessment of intra-atrial thrombosis (especially in the LAA; Figure 5), the evaluation of the mitral valve and in the performance of structural interventions, particularly in the hemodynamics laboratory, where LA is the treatment target (closure of the LAA, patent foramen ovale, etc.) or a step for treatment (mitraclip, periprosthetic leak treatment, etc.) (Figure 6) [37,38,39,40]. In the field of structural heart interventions, TEE has become the technique of choice during treatment to help guide those procedures that involve the LA in the performance of disease management [38]. Furthermore, TEE has demonstrated good correlation with TTE in the evaluation of atrial function and size, although it usually slightly underestimated the parameters in relation to the cavity size [11,41]. Nevertheless, because the probe is very close to the LA, in many cases, it is difficult to obtain the entire cavity in one plane, which does not allow for an overall assessment.

### 3.3. CMR

CMR allows assessment of LA morphology and functionality, as well as tissue characterization of the atrial wall, and is considered the gold standard for non-invasive study of atrial volumes [42,43].

**Figure 7:**
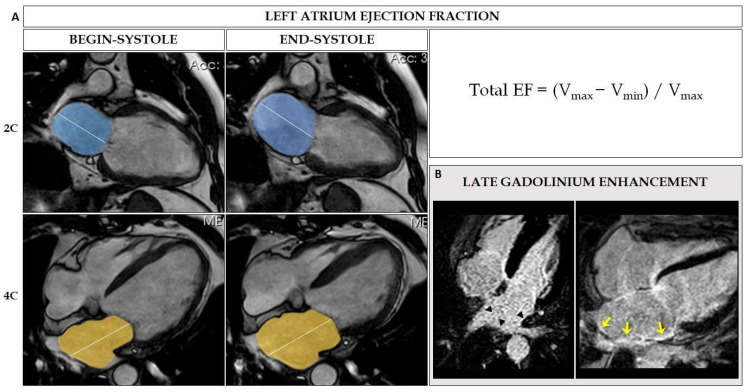
CMR sequences for LA evaluation. (**A**) LA volume and ejection fraction assessment, using two-chamber and four-chamber views, with areas and lengths planimetered (including the formula for the calculation of total ejection fraction). (**B**) Late gadolinium enhancement sequence to identify fibrosis around the PVs and in the LA wall prior pulmonary ablation procedure. (**Left Panel**) Arrowheads demonstrate absence of fibrosis in the atrial wall. (**Right Panel**) Yellow arrows indicate presence of fibrosis in the atrial wall.

Two-dimensional cine sequences are commonly employed to measure LA diameters and volumes and to evaluate global LA function (ejection fraction). The biplane area–length method (Figure 7), using two-chamber and four-chamber cine sequences, or the Simpson method, with a dedicated LA short-axis stack, have demonstrated a good correlation with 3D volumes without the necessity of contrast administration [44,45].

Three-dimensional CMR angiography sequences are a very accurate method to assess LA shape, LA dimensions, and the antrum of the PVs. These sequences also permit the identification of common anatomical variants such as a right intermediate pulmonary vein or a left common trunk [44]. In addition, three-dimensional CMR angiographic images can be exported to electroanatomical navigation systems, obtain fusion images, and thus make radiofrequency applications faster and easier. Recently, the feature-tracking technique has been developed, which allows a quantitative assessment of global and regional myocardial function to be obtained from the quantification of myocardial deformation parameters [46]. With LA measurements by CMR feature-tracking sequences, it is feasible to obtain the analysis of atrial strain in its different phases in a similar approach as TTE [11].

However, the fundamental interest in CMR lies in the detection and quantification of atrial fibrosis non-invasively (Figure 7) [44]. High-resolution late gadolinium enhancement sequences with free breathing with navigators have been designed to obtain information from the atrial wall (approximately 3 mm). This technique allows the identification of fibrosis around the PVs and in the LA wall after pulmonary ablation procedures, but also native LA fibrosis in other illnesses [47,48].

CMR has also been demonstrated to be useful in the characterization of intra-atrial masses, e.g., tumors such as myxoma or intracavitary thrombi [49]. However, its ability to detect these masses is considerably reduced when they are small and highly mobile, due to the limited temporal resolution of the technique.

### 3.4. MDCT

**Figure 8:**
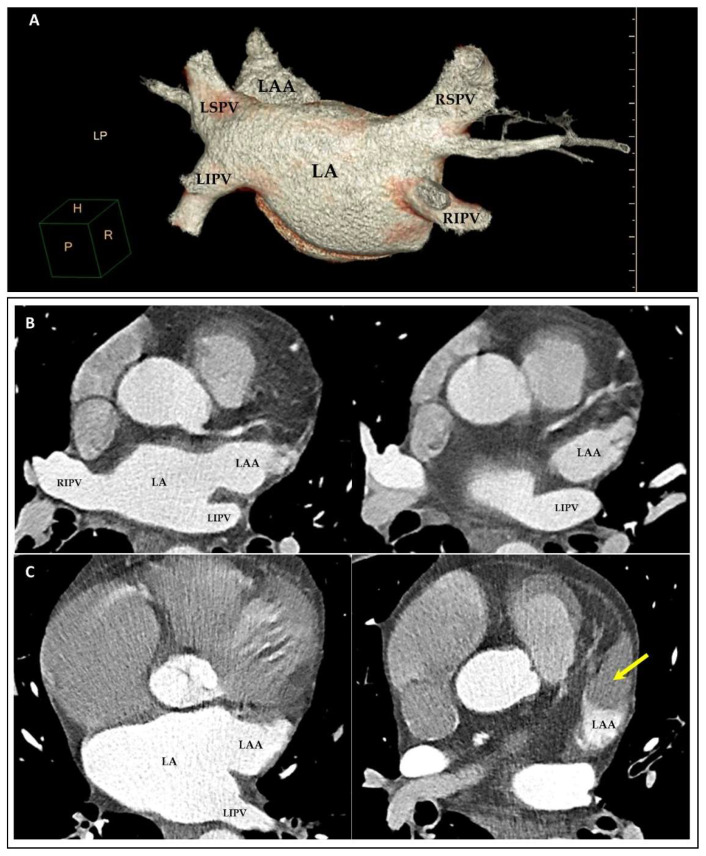
MDCT images focusing on the LA and the anatomical relationships. (**A**) Volume-rendered image showing LA and PVs prior to the performance of PV ablation in AF. (**B**,**C**) Axial MDCT images depicting LA and LAA prior to its closure. Yellow arrow in (**C**) shows presence of LAA thrombus. LA: left atrium; LAA: left atrial appendage; LIPV: left inferior pulmonary vein; LSPV: left superior pulmonary vein; RIPV: right inferior pulmonary vein; RSPV: right superior pulmonary vein.

Because of the high spatial resolution of the technique and the fast acquisition of the images, the study of the LA by MDCT focuses mainly on the anatomical assessment of the atrium [50]. Evaluation of the PVs prior to the performance of PV ablation in AF and the study of the LAA prior to its closure are two of the main indications of the technique (Figure 8) [51,52].

MDCT has also demonstrated to be useful in the calculation of atrial volumes and function with a good correlation with TTE [53]. However, the requirement of contrast administration and heart rate control medications in these cases, as well as the necessity to acquire protocols that include the entire atrial cycle, with the consequent increase in radiation for the study, makes the evaluation of atrial function a relatively infrequent indication of the technique.

MDCT has proven to be useful in the detection of thrombi, especially in LAA [54]. Nevertheless, although its sensitivity for detecting slowed flow or large thrombi is high, it is considerably lower for detecting smaller thrombi that are highly mobile.

Finally, it is also a suitable tool for the study of intra-auricular masses, as it provides information on the location and morphology of the lesion, but also on tissue characteristics and the contrast uptake type [50]. However, as these are relatively infrequent pathologies, this indication is uncommon in clinical practice.

## 4. Electroanatomical Mapping of the LA

Cardiac electroanatomical mapping is based on catheter navigation systems that are capable of displaying the three-dimensional (3D) position of electrophysiology catheters, as well as displaying cardiac electrical activity as waveform traces and as dynamic isopotential maps of the cardiac chamber. The evolution of the mapping system and catheter technology contributes to diagnosis and interventional treatment of cardiac arrhythmias. The cardiac mapping system provides a safe and accurate reconstruction of cardiac structures and a fast visualization of cardiac electrical circuits, allowing therapeutic decision making in invasive electrophysiology. These systems also reduce the amount of fluoroscopy time and radiation increasing safety for patients and staff [55].

### 4.1. Technique of Maps Acquisition

**Figure 9:**
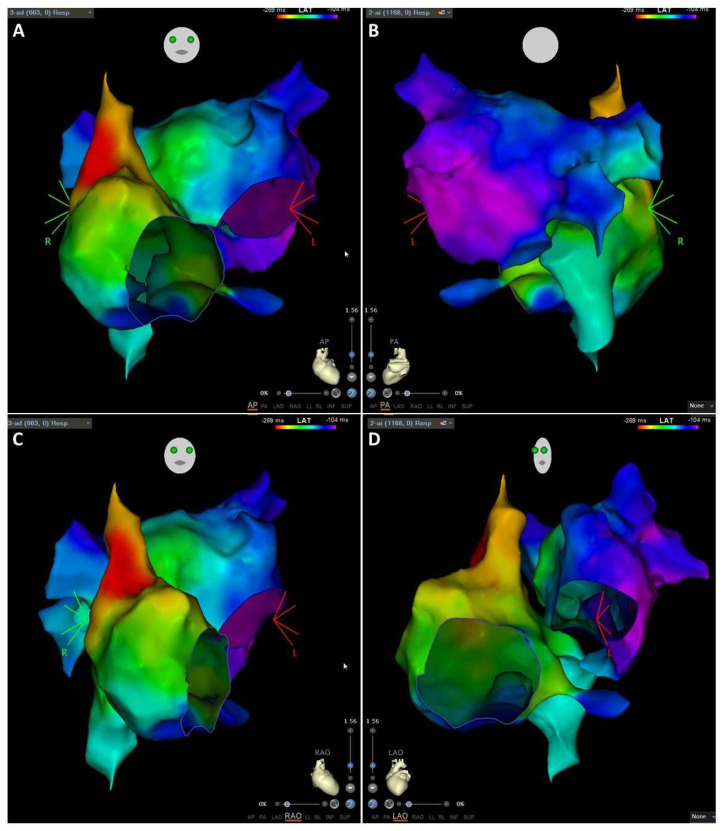
Electroanatomical map of a patient in normal sinus rhythm. Electroanatomical map of activation times of the right and the LA, performed during normal sinus rhythm. The atrial anatomy was determined by the magnetic catheter localization system integrated into the CARTO system. Activation sequence was derived from semi-automated sequential analysis of local electrograms. Activation times are color-coded with red indicating sites with the earliest activation and dark blue sites with the latest activations. (**A**,**B**): 2 images of the same map are shown in anteroposterior (AP) and posterior anterior (PA) projections, respectively. (**C**,**D**): 2 images of the same map are shown in right anterior oblique (RAO) and left anterior oblique (LAO) projections, respectively. Note the progressive red coding as the earliness of the mapped points increases relative to the onset of activation.

The navigation system can be used to locate one or more electrophysiology catheters in the heart and the contoured surfaces of the maps are based on the anatomy of the patient’s own cardiac chamber. During mapping, the clinician samples various heart locations by points in a stable rhythm using electrophysiology catheters. The mapping tool organizes data collected and the location of each point which is saved along with voltage and activation data, and finally can be displayed on the nearest surface as color and shows the data in 3D maps (Figure 9).

The EnSite Precision™ Cardiac Mapping System will collect impedance-based (NavX) points and magnetic-based (NavX SE) points and supports automatic high-resolution mapping. During model collection, both points are collected from a sensor tool. Field scaling can then be applied using either dataset to optimize the model and adjusts the dimensions of the navigation field based on both the position and orientation of magnetically located sensors and the electrodes on sensor tools. During ablation and for better lesion assessment, EnSite Precision works with several indices providing feedback about lesion quality. The combination of several pieces of information to a lesion index, which is composed of contact force, radiofrequency application duration, and radiofrequency current, has further improved the quality of lesion formation [56].

The CARTO system works with three separate low-level magnetic fields, uses hybrid current-based and magnetic information for catheter tracking and allows the system to create a precise electroanatomic map in real time with high spatial resolution. The contact force in the catheters is based on distance change between the magnetic transmitter coil and three location sensors with a known spring constant [57].

### 4.2. Types of Electroanatomical Maps: A Single Set of Collected Data Can Be Used to Display Several Types of Maps

**Figure 10:**
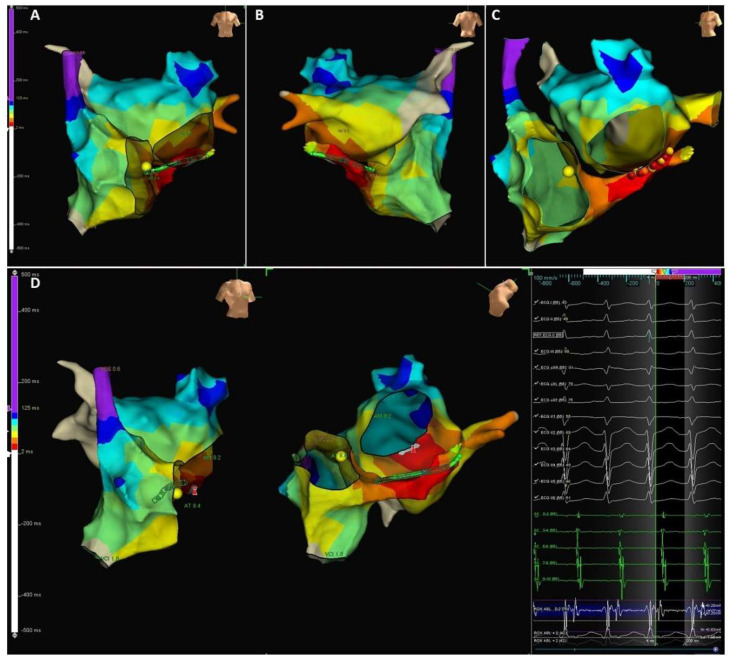
Supraventricular tachycardia due to left accessory pathway activation map. Electroanatomic map of activation times of the right and the LA, performed during supraventricular tachycardia due to concealed accessory left pathway. The atrial anatomy was determined by the impedance catheter localization system integrated into the NAVEx system. Activation sequence was derived from semi-automated sequential analysis of local electrograms. Activation times are color-coded with red indicating sites with earliest activation and purple sites with latest activations. (**A**–**C**): 3 images of the same map are shown in anteroposterior (AP) posterior anterior (PA) and caudal left anterior oblique (LAO) projections, respectively. (**D**): 2 images of the same map are shown in right anterior oblique (RAO) and caudal projections, respectively. The earliest site is the posterolateral part of mitral valve.

**Figure 11:**
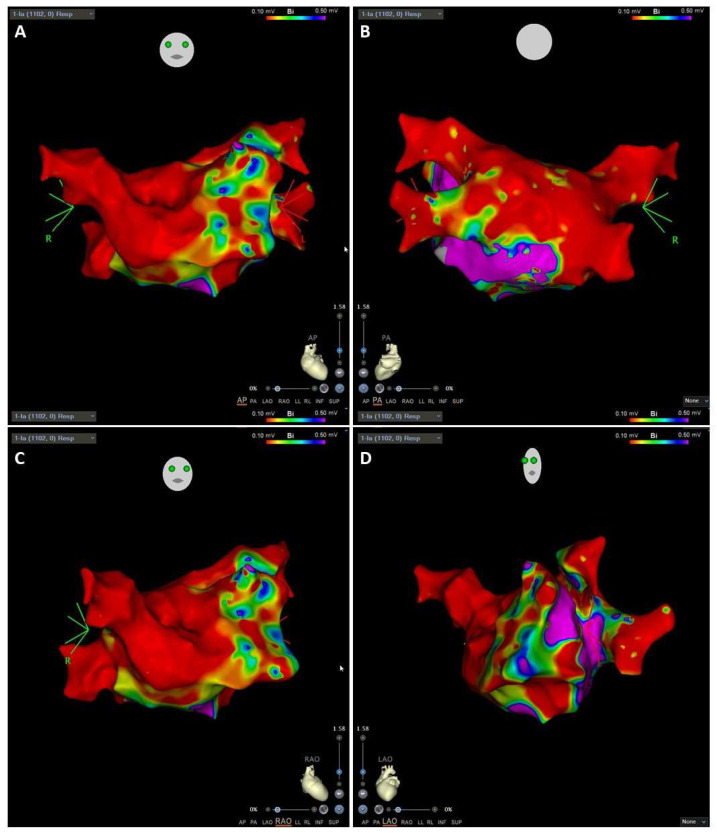
Substrate voltage map of LA in a patient with AF. Substrate voltage map of bipolar electrograms in the LA, performed in a patient with AF. The zones with voltage >0.5 mV identify normal tissue and are shown in purple and the red zone denotes voltage <0.1 mV and identify endocardial scar. Green, yellow, and blue represent intermediate voltages. Three-dimensional (3D) position coordinates were recorded with CARTO 3 (Biosense Webster, Diamond Bar, CA, USA) and a 20-pole circular mapping catheter. Peak-to-peak voltages were collected from the 10 dipoles and a high-density bipolar voltage map was created. Left atrial voltage and activation maps were constructed during SR before ablation. (**A**,**B**): 2 images of the same map are shown in anteroposterior (AP) posterior anterior (PA) projections, respectively. (**C**,**D**): 2 images of the same map are shown in right anterior oblique (RAO) and left anterior oblique (LAO) projections, respectively.

Cardiac Triggered Maps: Use a surface electrocardiogram or an intracardiac electrogram as the reference to which collected points are measured. The most frequently employed are:

- Local Activation Time isochronal maps display color-coded activation times for each collected location. The local activation time is the difference in milliseconds between detected activation on the roving waveform and the reference waveform (Figure 10). Reentrant arrhythmias differ in conduction velocity and refractory period. In accessory pathways, atrial impulses can travel through the atrioventricular node or through these abnormal pathways and may produce an excessive increase in the ventricular rate. The definitive treatment is the destruction of the electrical pathway by catheter ablation [58].
- Voltage maps or Substrate maps display color-coded voltage values for each collected location. The voltage is the difference in millivolts between the components of the detected activation complex on the roving waveform (Figure 11). Transition from paroxysmal to non-paroxysmal AF is often characterized by advancing atrial structural remodeling or worsening of atrial cardiomyopathy. During AF, the atria undergo electrophysiological and structural changes which promote the progression of AF. Among these changes, the accumulation of fibrosis is a key factor in determining a patient’s susceptibility to arrhythmias [9].

Non-Cardiac Triggered Maps: Collect points at one second intervals.

- Complex Fractionated Electrogram Mean maps provide a fractionation index based on the cycle length between multiple, discrete, local activations in an intracardiac electrogram. Collected points with a lower value are mapped toward the white end of the color spectrum.

## 5. Conclusions

Cardiac imaging techniques have developed multiple tools for LA assessment in recent years. Although TTE is the most widely employed procedure for the calculation of atrial size and function, due to its ease of use and wide availability, CMR, MDCT and TEE are also options for its study. In particular, CMR is considered the most accurate technique for assessing atrial volumes. In addition, MDCT and CMR are capable of generating 3D anatomical images very useful for the management of cardiac rhythm disorders of atrial origin. CMR permits estimation of the amount and location of fibrosis in the atrial wall using late gadolinium enhancement sequences, helping in the treatment of atrial arrhythmias, and opens up a potential field of study in many other pathologies. TEE assists in the evaluation of specific atrial structures, especially in the preparation and performance of invasive treatments such as LAA or patent foramen ovale closure. This technique is essential in the hemodynamics laboratory to guide invasive procedures. Electroanatomical mapping in the electrophysiology laboratory gives us an electrical view of the LA that provides a more accurate treatment of arrhythmias generated in the atrium. Thus, although LA assessment should be performed in all routine cardiac imaging studies, the use of multimodality is mandatory to address the most appropriate treatment in diseases of primarily atrial origin.

## Figures and Tables

**Table 1:** Reference ranges of LA dimension and function by 2- and 3-dimensional echocardiography.

Parameter	Formula	Normal Range (Values 2D)	Normal Range (Values 3D)
**Left Atrial Size**			
**Maximal AP Diameter, mm** [23]	In parasternal long axis view in telesystole	Male: 30–40 Female: 27–38	
**Maximal Area, cm^2^/m^2^** [23]	In apical 4 chamber view	Male: 8.9 ± 1.5 Female: 9.3 ± 1.7	
**Left Atrial Functional Parameters By 2D and 3D Echocardiography**			
**Vmax (mL/m^2^) ^1^** [24]	Before mitral valve opening in 4Ch	25.7 ± 7.9	28.1 ± 6.9
**Vmin (mL/m^2^) ^1^** [24,25]	In the onset the P wave in 4Ch	8 ± 3	10.7 ± 3.7
**VPreA (mL/m^2^) ^1^** [24,25]	Before mitral valve closure in 4Ch	15 ± 5	17.8 ± 5.5
**Total EV (mL) ^1^** [24,25]	Vmax − Vmin	29 ± 7	30.9 ± 9.0
**Passive EV (mL) ^1^** [24,25]	Vmax − VpreA	17 ± 6	18.4 ± 6.4
**Active EV (mL) ^1^** [25]	preA-Vmin	12 ± 4	14 ± 6
**Total EF (%) ^1^** [24,25]	[(Vmax − Vmin)/Vmax] × 100%	69 ± 9	62.2 ± 7.7
**Passive EF (%) ^1^** [24,25]	[(Vmax − Vpre-A)/Vmax] × 100%	41 ±10	37.7 ± 11.0
**Active EF (%) ^1^** [24,25]	[(VpreA −Vmin)/VpreA] × 100%	47 ± 10	39.5 ± 9.5
**Expansion Index, % ^2^** [25]	[(Vmax − Vmin)/Vmin] × 100%	204 (165; 289)	208 (171; 250)
**Left Atrial Phasic Function**			
**Reservoir LS (%)** [26]	R wave as time reference	39.4% (95% CI, 38.0–40.8%)	
**Conduit LS (%)** [26]	R wave as time reference	23.0% (95% CI, 20.7–25.2%)	
**Contractile LS (%)** [26]	R wave as time reference	17.4% (95% CI, 16.0–19.0%)	

^1^ Values are reported as mean ± SD. Vmax: maximum volume. Vmin: minimum volume. VPreA: preA volume. EV: emptying volume. EF: ejection fraction. LS: longitudinal strain. ^2^ Values are reported as median (25th percentile; 75th percentile).

## Data Availability

No new data were created or analyzed in this study. Data sharing is not applicable to this article.

## Abbreviations

AF	Atrial fibrillation
CMR	Cardiac magnetic resonance
LA	Left atrium
LAA	left atrial appendage
LV	Left ventricle
MDCT	Multidetector computed tomography
PVs	Pulmonary veins
TEE	Transesophageal echocardiography
TTE	Transthoracic echocardiography
